# Blended Care Therapy for Depression and Anxiety: Outcomes across Diverse Racial and Ethnic Groups

**DOI:** 10.1007/s40615-022-01450-z

**Published:** 2022-12-02

**Authors:** Jocelynn T. Owusu, Pam Wang, Robert E. Wickham, Danielle P. Cottonham, Alethea A. Varra, Connie Chen, Anita Lungu

**Affiliations:** 1 Lyra Health, Burlingame, CA USA; 2 Department of Psychological Sciences, Northern Arizona University, Flagstaff, AZ USA

**Keywords:** Depression, Anxiety disorders, Culturally responsive care, Internet-based interventions

## Abstract

**Background:**

Studies have reported positive outcomes of blended care therapy (BCT), which combines face-to-face care with internet modules. However, there is insufficient evidence of its effectiveness across racial and ethnic groups. This study evaluated outcomes of a BCT program, which combined video psychotherapy with internet cognitive-behavioral modules, across race and ethnicity.

**Methods:**

Participants were 6492 adults, with elevated anxiety (Generalized Anxiety Disorder-7 [GAD-7] ≥ 8) and/or depression (Patient Health Questionnaire-9 [PHQ-9] ≥ 10) symptoms, enrolled in employer-offered BCT. Changes in anxiety (GAD-7) and depression (PHQ-9) symptoms during treatment were evaluated using individual growth curve models. Interaction terms of time with race and ethnicity tested for between-group differences. Treatment satisfaction was assessed using a Net Promoter measure (range = 1 (lowest satisfaction) to 5 (greatest satisfaction)).

**Results:**

Participants’ self-reported race and ethnicity included Asian or Pacific Islander (27.5%), Black or African American (5.4%), Hispanic or Latino (9.3%), and White (47.2%). Anxiety symptoms decreased during treatment (*p* < 0.01), with greater reductions among Hispanic or Latino participants compared to White participants (*p* < 0.05). Depressive symptoms decreased across treatment (*p* < 0.01), with significantly greater decreases among some racial and ethnic groups compared to White participants. Declines in anxiety and depressive symptoms slowed across treatment (*p*’s < 0.01), with statistically significant differences in slowing rates of depressive symptoms across some racial and ethnic groups. Among participants with responses (28.45%), average treatment satisfaction ranged from 4.46 (SD = 0.73) to 4.67 (SD = 0.68) across race and ethnicity (*p* = 0.001). Racial and ethnic differences in outcomes were small in magnitude.

**Conclusions:**

BCT for anxiety and depression can be effective across diverse racial and ethnic groups.

**Supplementary Information:**

The online version contains supplementary material available at 10.1007/s40615-022-01450-z.

## Introduction

Depression and anxiety are common mental disorders in the USA and worldwide [1]. Efficacious psychotherapies like cognitive behavioral therapy (CBT) exist to treat these conditions [2, 3]; however, many adults with depression and anxiety do not receive mental health care [4, 5]. This disproportionately impacts several communities of color who access care at lower rates compared to White adults [4, 6]. In the USA, the prevalence of depression and anxiety symptoms increased threefold during the COVID-19 pandemic relative to before the pandemic [7–9], with some communities of color experiencing a greater burden than White adults [9, 10]. Now, more than ever, effective and accessible mental health services are essential to address mental health care needs across diverse populations.

Because of their greater scalability, telehealth, and internet-based psychological interventions have the potential to mitigate accessibility barriers to traditional psychotherapy [11, 12] and provide more accessible care to communities of color that experience mental health disparities [13, 14]. It is essential such programs demonstrate effectiveness and equitable outcomes across racial and ethnic groups when delivered at a meaningful scale. Yet, outcomes research of internet-based mental health interventions has primarily been conducted in samples that under-represent communities of color [14], and racial and ethnic differences in clinical outcomes of these interventions are not commonly evaluated. Studies of tele-counseling interventions and internet CBT (iCBT) have reported positive anxiety and depression treatment outcomes among communities of color [15–17]. However, most of this research has been limited by small sample sizes or has evaluated programs targeting specific racial and ethnic groups, increasing the difficulty in fully grasping the impact of large-scale interventions that aim to successfully treat diverse populations.

These research gaps also extend to blended care therapy (BCT), another more scalable treatment for anxiety and depression. BCT combines face-to-face psychotherapy sessions with digital content (e.g., lessons and exercises to be completed between therapy sessions) and has numerous potential benefits including reducing treatment dropout and improving psychotherapy outcomes [18]. Whereas, a recent review of over 40 studies supports the effectiveness of blended care interventions compared to controls not receiving treatment [18], BCT outcomes across racial and ethnic groups are not well understood. The lack of clarity around whether telehealth and internet-based psychological interventions are more effective for some racial and ethnic groups over others reinforces the need to evaluate the outcomes of such interventions across race and ethnicity [14].

In order to provide equitable mental health care across diverse populations, researchers have highlighted the need for incorporating cultural competency within both the organization and the direct services of mental health care providers [19, 20]. Such an approach aims to ensure that mental health providers and the care they offer are responsive to the unique cultural attributes of the communities served. This includes the availability of mental health services that address the care needs of the target community [19, 20]. These recommendations should be extended to internet-based psychological care, including telehealth, that aims to widen the reach of traditional psychotherapy and serves broader populations which include marginalized groups (e.g., communities of color). Telehealth, in particular, could facilitate access to culturally competent care which may have limited reach otherwise [21]. Culturally competent care can be further built upon by also incorporating culturally responsive approaches such as cultural humility into care delivery [22]. Unlike cultural competency which can suggest providers can reach competence in their ability to provide care within participants’ unique cultural contexts, culturally responsive care highlights that providing such treatment requires ongoing self-reflection and learning on the part of providers [23]. There is a shortage of large-scale telehealth and internet-based psychological interventions that are delivered with an emphasis on cultural competence or responsiveness, despite suggestions that incorporating cultural factors into such interventions could improve engagement and outcomes [14].

In order to address the described research gaps and generate further evidence of digital mental health care delivered with cultural responsiveness, the current study evaluated outcomes of a large-scale, employer-offered, culturally responsive blended care therapy (BCT) program, which combined live video-based psychotherapy sessions with therapist-assigned iCBT modules, across racial and ethnic groups. Specifically, this study assessed clinical outcomes (i.e., symptoms of depression and anxiety) across treatment, as well as end-of-care treatment satisfaction, in a large, multi-ethnic sample that included a significant proportion of participants from communities of color. This research expands upon a prior large-scale study of this BCT program that evaluated clinical outcomes without investigating potential racial and ethnic differences [24]. To the best of our knowledge, this study is the first in the USA to evaluate outcomes of a large-scale BCT program across diverse racial and ethnic groups.

## Methods

### Design and Participants

**Fig. 1:**
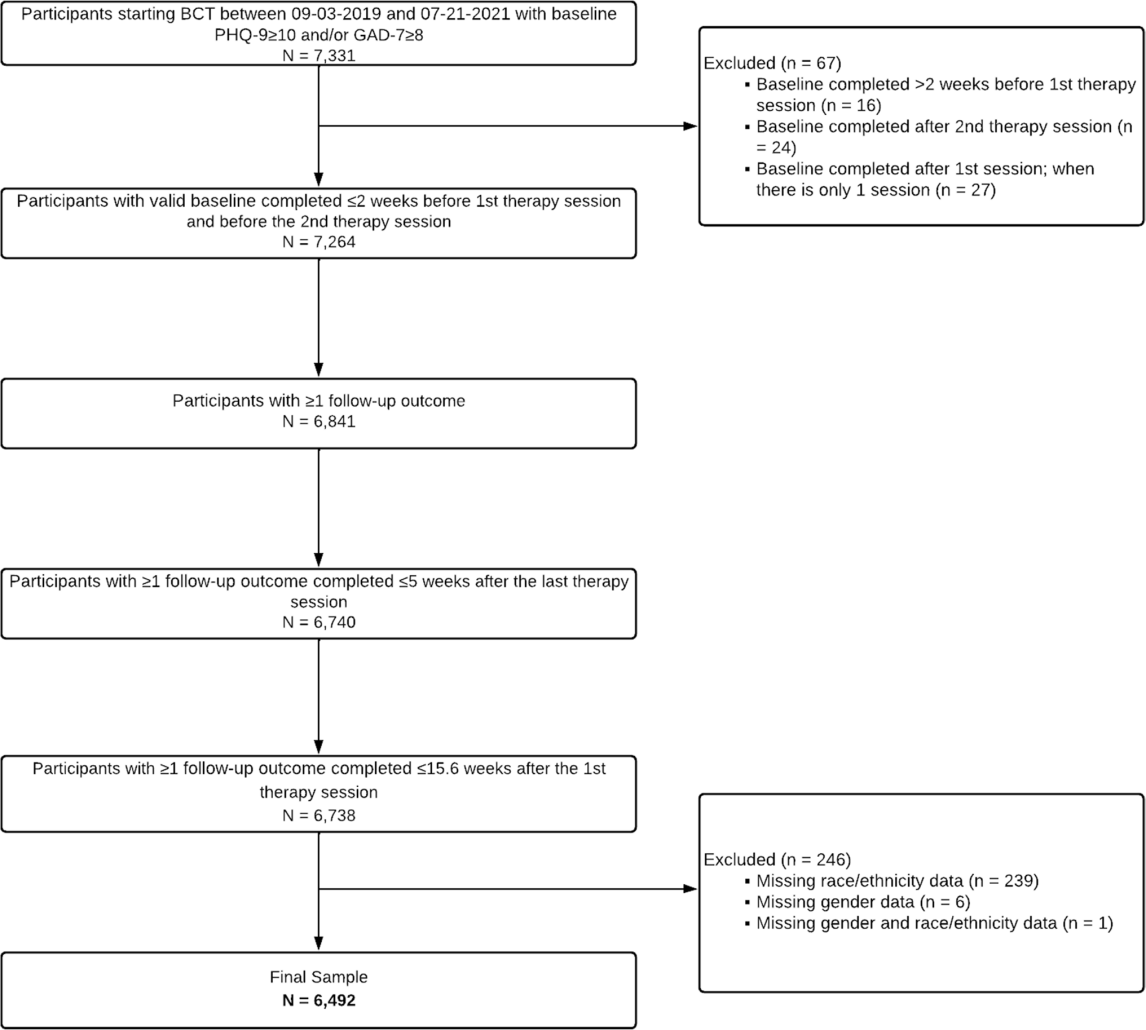
Participant flowchart. Notes. BCT: blended care therapy; PHQ-9: 9-item Patient Health Questionnaire; GAD-7: 7-item Generalized Anxiety Disorder Scale

This was a follow-up study of a pragmatic retrospective cohort analysis that included participants who received care between September 3rd, 2019 and July 21st, 2021 [24]. The BCT program evaluated in the current study was a mental health benefit offered by Lyra Health and its contracted partner, Lyra Clinical Associates, to employees, and their dependents, through their employer. Participants were employed by, or dependents of employees from, 77 companies covering a diverse array of industry sectors. All participants and therapists resided in the USA across all 4 regions (i.e., Northeast, Midwest, South, West) [25]. Adults aged ≥ 18 years who began the BCT program during the study period with elevated symptoms of anxiety (i.e., Generalized Anxiety Disorder-7 [GAD-7] score ≥ 8) [26] and/or depression (i.e., Patient Health Questionnaire-9 [PHQ-9] score ≥ 10) [27] upon registration, were eligible for the current study (*N* = 7,331). In order to minimize any potential influence of treatment on baseline assessment scores, participants were excluded if they did not have a valid baseline assessment that was collected within 2 weeks of the first therapy session and before the second therapy session. Because this study aimed to evaluate outcomes over the course of treatment and at the end of care, participants were also excluded if they did not have at least 1 valid follow-up assessment that was completed within 5 weeks of their final therapy session and within 15.6 weeks of the first therapy session (i.e., within 1 standard deviation of the average BCT program duration). Participants who did not self-report gender were also excluded. Finally, participants who opted not to disclose race and ethnicity, or were otherwise missing race and ethnicity data, were excluded. The final sample size was 6492 participants (88.56% of the eligible sample). Figure 1 depicts the participant study flowchart.

### Treatment

A previous publication provided a comprehensive description of the BCT program [28]. In sum, the BCT program merges synchronous video-based psychotherapy sessions with asynchronous digital tools that are implemented in-between live therapy sessions. Quality assurance included the use of an internal fidelity rating scale to assess the content of therapy sessions. Participants are recommended 5–10 therapists based on factors such as clinical fit and modality preference and self-select whom they ultimately want to work with. Additionally, as a part of the BCT onboarding process, participants have the option to search for therapists from communities of color who also have expertise in the area in which they are seeking support (e.g., racial stress).

Digital tools were established from transdiagnostic approaches to CBT, including Acceptance Commitment Therapy and Dialectical Behavior Therapy [29, 30]. These tools comprise lessons, exercises, and assessments. Digital lessons use a storytelling approach to follow the personal treatment journey of characters who are experiencing anxiety or depression symptoms.

The BCT program incorporates culturally responsive practices [22, 31, 32], which include providing culturally appropriate support across diverse racial and ethnic identities. From a clinical perspective, culturally responsive care (CRC) grounds evidenced-based practices in the values of cultural responsiveness, humility, and respect. The integration of these values into clinical practice is a skill that can be developed and strengthened over time. Thus, training and education are key components of CRC. As mentioned previously, CRC encompasses ongoing learning; therefore, the BCT program is continuously enhanced to increase its cultural responsiveness. Because it is essential that culturally responsive blended care fuses culturally responsive approaches into both the psychotherapy and digital tools, these approaches are summarized below.

#### Culturally responsive therapists

BCT providers complete a webinar on core CRC concepts, such as intersectionality and power and privilege within the therapeutic relationship [33, 34]. Additionally, therapists have access to individual and group consultation with specialists in culturally responsive care, and continuing education training that builds upon core culturally responsive care concepts. CRC is also incorporated into measuring the quality of care with the BCT program. For example, therapists’ level of cultural attunement and responsiveness is a component of the vetting process and overall clinical performance. In addition, quality assurance includes regular assessment of therapeutic alliance. The on-going consultation support clinical supervisors offer their providers plays a significant role in client care as it is an opportunity for providers to strengthen their clinical skills. For this reason, clinical supervisors also receive similar training and consultation support in CRC focused primarily on enhancing culturally responsive supervision skills.

#### Culturally responsive digital tools

Culturally responsive care is also integrated into the development of digital tools available in the platform. For example, BCT includes digital lessons and handouts on coping with and responding to racial microaggressions [35–38]. Specific digital tools are assigned by therapists to ensure they are aligned with clients’ unique case formulation and presenting concerns for therapy [28]. In addition, all digital content is reviewed with specific attention given to the representation of characters from communities of color in the storylines and to the language used in sharing the experiences of these characters. Content reviews also aim to limit the perpetuation of problematic stereotypes rooted in racial bias.

## Study Measures

As a part of BCT, participants’ anxiety and depressive symptom severity were monitored via weekly administration of PHQ-9 and GAD-7 scales. A PHQ-9 score ≥ 10 indicates clinically elevated depressive symptomatology [27], and GAD-7 scores ≥ 8 indicate clinically elevated anxiety symptomatology [26]. Among those with clinically elevated symptoms at baseline, recovery was defined as a reduction in scores below the clinical threshold of 10 on the PHQ-9 and/or 8 on the GAD-7 by the final assessment. Meanwhile, among these participants, reliable improvement was defined as a reduction of 4 or more on the GAD-7 and/or 6 or more on the PHQ-9 by the final assessment. Reliable improvement score cutoffs were derived from reliable change indices calculated for the PHQ-9 and GAD-7 scales in prior research [39, 40].

Self-reported treatment satisfaction was collected after participants’ final BCT session, using the following Net Promoter measure [41]: “How likely are you to recommend your Lyra therapist to someone with needs similar to yours?”. In this study, responses were coded as: “Extremely unlikely” = 1, “Unlikely” = 2; “Neutral” = 3; “Likely” = 4; “Extremely likely” = 5. This item was averaged, and higher scores indicated greater satisfaction with BCT.

Along with additional demographic data, race and ethnicity were collected from BCT participants for the purpose of evaluating treatment quality and outcomes across demographic groups. Participants self-reported race and ethnicity during the client intake and registration. Participants reported whether they identified as “American Indian or Alaska Native,” “Asian or Pacific Islander,” “Black or African American,” “Hispanic or Latino,” “Native Hawaiian or Other Pacific Islander,” “White,” “Prefer Not to Disclose,” and/or “Other.” Participants who selected more than one race or ethnicity (e.g., those who selected both “White” and “Hispanic or Latino”, etc.) were re-categorized as “Multiple.” In addition, those who reported “American Indian or Alaska Native,” “Native Hawaiian or Other Pacific Islander,” or “Other” were re-categorized as “Other” due to small sample sizes. As mentioned previously, participants who selected “Prefer Not to Disclose” or did not respond to this item were excluded from this study. Self-reported highest educational attainment was re-categorized “College Degree or Higher” (i.e., “College graduate or above”) versus “No College Degree” (i.e., “Some college or AA degree,” “High school graduate/GED or equivalent,” “9-11th grade (Includes 12th grade with no diploma),” “Less than 9th grade”). Participants also rated their financial status (i.e., “Good,” “Fair,” “Poor”), in addition to self-reporting age and gender.

## Statistical Analysis

Potential differences in mean satisfaction scores, duration in treatment, and number of psychotherapy sessions across race and ethnicity were tested using Analysis of Variance (ANOVA) with Bonferroni-corrected follow-up comparisons [42]. In order to assess end-of-care treatment response, rates of reliable improvement, recovery, reliable improvement and recovery, and reliable improvement or recovery in symptoms of anxiety or depression were calculated. Racial and ethnic differences in these outcomes were assessed using *χ*^2^ tests. To assess outcomes over the course of care, changes in weekly anxiety and depression symptoms throughout the treatment course were modeled using individual growth curve (IGC) analysis [43]. Participants’ responses to anxiety (GAD-7) and depression (PHQ-9) symptom measures were modeled as non-linear IGCs by incorporating linear (Week) and quadratic (Week^2^) time indicators as fixed effects. Random effects for the intercept term were estimated at the participant and therapist levels, along with random effects for the linear and quadratic effects at the participant level. Potential differences in initial symptom levels across race and ethnicity were evaluated by adding race and ethnicity categories as predictors, with White participants as the reference group. Differences in the magnitude of the initial decline in symptoms were evaluated by incorporating interaction terms between each race and ethnicity predictor and the (linear) Time variable, whereas potential differences in the tapering of initial decline were modeled by including interaction terms between each race and ethnicity predictor and the Time^2^ variable. Because therapy sessions were the most fundamental component of the BCT program, the final models also incorporated time-varying covariate effects of engaging with a video therapy session during the past week (last 7 days) or prior week (8–14 days). Potential differences in the magnitude of these time-varying covariate coefficients across race and ethnicity categories were also evaluated by incorporating the corresponding interaction terms. For each outcome, models were also fit that included age and gender as fixed effects. Because the addition of these variables did not significantly improve model fit, they were excluded from the final models. However, Supplemental Tables 1 and 2 report both the final models and models that include age and gender as fixed effects.

## Results

Participant characteristics of the final sample are provided in Table 1 (*N* = 6492). Most participants were White adults (*n* = 3065; 47.21%), followed by Asian or Pacific Islander adults (*n* = 1785; 27.50%). On average, participants were aged 33.19 years (SD = 8.75), and the majority were female (*n* = 4239; 65.30%). Most participants had attained a college degree or higher (*n* = 5185; 79.87%), and a large proportion self-reported their financial status as “good” (*n* = 4,012; 61.80%) or “fair” (*n* = 2099; 32.33%).

**Table 1:** Participant characteristics across race and ethnicity, mean (SD) or column %’s

	Entire sample *N* = 6492 (100%)	Asian or Pacific Islander *n* = 1785 (27.50%)	Black or African American *n* = 351 (5.41%)	Hispanic or Latino *n* = 601 (9.26%)	Multiple *n* = 532 (8.19%)	Other *n* = 158 (2.43%)	White *n* = 3065 (47.21%)	F or *χ*^2^ *p-value*
Age, mean (SD)	33.19 (8.75)	31.49 (7.09)	33.75 (9.08)	31.88 (7.68)	31.43 (7.92)	33.18 (7.96)	34.67 (9.64)	*F* (5,6486) = 39.6 *p* < 0.001
Gender, *n* (%)								
Female	4239 (65.30)	1178 (65.99)	246 (70.09)	384 (63.89)	371 (69.74)	90 (56.96)	1,970 (64.27)	*χ*^2^ = 15.34 *p* = 0.009
Male	2253 (34.70)	607 (34.01)	105 (29.91)	217 (36.11)	161 (30.26)	68 (43.04)	1095 (35.73)	
Educational attainment, *n* (%)								
College Degree or Higher	5185 (79.87)	1559 (87.34)	224 (63.82)	378 (62.90)	382 (71.80)	136 (86.08)	2506 (81.76)	*χ*^2^ = 338.61^a^ *p* < 0.001
No College Degree	1125 (17.33)	138 (7.73)	117 (33.33)	213 (35.44)	138 (25.94)	20 (12.66)	499 (16.28)	
*Missing*	*182 (2.80)*	*88 (4.93)*	*10 (2.85)*	*10 (1.66)*	*12 (2.26)*	*2 (1.27)*	*60 (1.96)*	
Financial status, *n* (%)								
Good	4012 (61.80)	1098 (61.51)	133 (37.89)	269 (44.76)	308 (57.89)	101 (63.92)	2103 (68.61)	*χ*^2^ = 287.04^b^ *p* < 0.001
Fair	2,099 (32.33)	600 (33.61)	169 (48.15)	275 (45.76)	184 (34.59)	50 (31.65)	821 (26.79)	
Poor	273 (4.21)	28 (1.57)	42 (11.97)	50 (8.32)	33 (6.20)	7 (4.43)	113 (3.69)	
*Missing*	*108 (1.66)*	*59 (3.31)*	*7 (1.99)*	*7 (1.16)*	*7 (1.32)*	*0*	*28 (0.91)*	
Baseline GAD-7 ≥ 8, *n* (%)	5,812 (89.53)	1,601 (89.69)	311 (88.60)	551 (91.68)	474 (89.10)	142 (89.87)	2,733 (89.17)	*χ*^2^ = 3.89 *p* = *0.57*
Baseline mean (SD)^c^	12.66 (3.51)	12.60 (3.49)	13.02 (3.66)	13.07 (3.62)	12.52 (3.49)	12.66 (3.63)	12.60 (3.47)	*F* (5, 5806) = 2.60 *p* = 0.02
Baseline PHQ-9 ≥ 10, *n* (%)	3,802 (58.56)	1,064 (59.61)	222 (63.25)	376 (62.56)	328 (61.65)	90 (56.96)	1,722 (56.18)	*χ*^2^ = 17.36 *p* = *0*.*004*
Baseline mean (SD)^d^	14.07 (3.54)	13.98 (3.50)	14.82 (3.88)	14.29 (3.65)	14.30 (3.66)	13.89 (3.69)	13.94 (3.45)	*F* = 3.16 (5, 3796) *p* = 0.008

*SD* standard deviation. ^a^ Participants missing data on educational attainment were excluded from *χ*^2^ test. ^b^ Participants missing data on financial status were excluded from *χ*^2^ test. ^c^ Calculated among participants with a baseline GAD-7 ≥ 8 only; ^d^ Calculated among participant with a baseline PHQ-9 ≥ 10 only

Overall, 89.53% of the sample reported baseline GAD-7 scores above the clinical threshold (≥ 8), and there were no significant differences across racial and ethnic groups (*χ*^2^(5) = 3.89, *p* = 0.57, φ = 0.07). Among participants with a baseline GAD-7 ≥ 8, a one-way ANOVA found significant (albeit small) cross-group differences among mean baseline GAD-7 scores (*F* (5, 5806) = 2.60, *p* = 0.02, adjusted *R*^2^ = 0.0001). However, post-hoc pairwise independent sample t-tests did not find statistically significant differences between any two racial and ethnic groups after performing Bonferroni corrections. Meanwhile, 58.56% of the sample reported baseline scores within the clinical range on the PHQ-9 (≥ 10), and there were statistically significant differences across racial and ethnic groups (*χ*^2^ (5) = 17.36, *p* = 0.004, φ = 0.03). Among participants with a baseline PHQ-9 ≥ 10, there were also significant cross-group differences in mean PHQ-9 scores at baseline (*F* (5, 3796) = 3.16, *p* = 0.008, adjusted *R*^2^ = 0.003). However, in post-hoc tests, there was only a statistically significant difference in baseline PHQ-9 scores between Black or African American and White participants after performing Bonferroni corrections (Mean baseline PHQ-9 scores = 14.82 versus 13.94, respectively; *p* = 0.02).

### Treatment Duration

Treatment duration for the overall sample and across race and ethnicity are provided in the top panel of Table 2. On average, participants attended 6.01 therapy sessions (SD = 3.55) and were under care for 7.61 weeks (SD = 6.21). There were significant differences in the number of therapy sessions (*F* (5, 6486) = 3.76, *p* = 0.002) across race and ethnicity, but not the duration of care (*F* (5, 6486) = 1.84, *p* = 0.10, adjusted *R*^2^ < 0.001). The magnitude of the differences in the mean number of therapy sessions across racial and ethnic groups was trivial (adjusted *R*^2^ = 0.002). In post-hoc tests, Asian or Pacific Islander adults completed significantly fewer sessions (mean = 5.76, SD = 3.37) than those who reported multiple racial or ethnic groups (mean = 6.28, SD = 3.60) and White adults (mean = 6.12, SD = 3.57) after Bonferroni corrections (*p*’s = 0.04 and 0.006, respectively).

**Table 2:** Treatment duration and satisfaction scores across race and ethnicity

	Entire sample	Asian or Pacific Islander	Black or African American	Hispanic or Latino	Multiple	Other	White	*F*-statistic *p-value*	Adjusted *R*^2^
Treatment duration	*N* = 6492	*n* = 1785	*n* = 351	*n* = 601	*n* = 532	*n* = 158	*n* = 3065		
# of Therapy sessions completed, mean (SD)	6.01 (3.55)	5.76 (3.37)	5.68 (3.78)	6.09 (3.70)	6.28 (3.60)	6.16 (3.71)	6.12 (3.57)	3.76 *p* = 0.002	0.002
Duration of care (in weeks), mean (SD)	7.61 (6.21)	7.29 (6.04)	7.43 (6.56)	7.75 (6.53)	8.05 (6.50)	8.10 (7.05)	7.68 (6.10)	1.84 *p* = 0.10	0.0006
Satisfaction scores,^a^ *n* (%)^b^	*n* = 1847 (28.45%)	*n* = 514 (28.80%)	*n* = 100 (28.50%)	*n* = 158 (26.30%)	*n* = 148 (27.82%)	*n* = 46 (29.11%)	*n* = 881 (28.74%)		
Mean (SD)	4.57 (0.73)	4.46 (0.73)	4.56 (0.86)	4.67 (0.68)	4.66 (0.67)	4.57 (0.69)	4.61 (0.73)	4.07 *p* = 0.001	0.008
Median	5	5	5	5	5	5	5		

^a^Item asked: “How likely are you to recommend your Lyra therapist to someone with needs similar to yours?” ^b^Sample size and proportion of participants with satisfaction score data by race and ethnicity

### Treatment Satisfaction

In total, 1847 (28.45%) participants self-reported treatment satisfaction (Table 2). There were no significant differences in the likelihood of responding to the satisfaction question (*χ*^2^ (5) = 1.75, *p* = 0.88, φ = 0.02) across racial and ethnic groups. However, independent samples *t*-tests suggested that responders reported significantly lower scores on the final GAD-7 (M_GAD-7_ = 4.13, SD_GAD-7_ = 3.73) and PHQ-9 (M_PHQ-9_ = 3.73, SD_PHQ-9_ = 3.92), relative to those who did not respond (M_GAD-7_ = 5.96, SD_GAD-7_ = 4.14; M_PHQ-9_ = 5.39, SD_PHQ-9_ = 4.58; both *p*’s < 0.001). The effect sizes of these differences were large (PHQ-9 & GAD-7 Hedges’ *g* = 1.46 and 1.76, respectively). Among responders, 92.10% were “extremely likely” or “likely” to recommend their Lyra therapist to someone with similar needs, and the mean score was 4.57 (SD = 0.73). ANOVA revealed statistically significant differences across racial and ethnic groups for satisfaction responses (*F* (5, 1841) = 4.07, *p* = 0.001), though these differences were small in magnitude, accounting for < 1% of the total variance in responses (adjusted *R*^2^ = 0.008). In post-hoc tests, Asian or Pacific Islander adults had significantly lower scores (mean = 4.46, SD = 0.73) than Hispanic or Latino adults (mean = 4.67, SD = 0.68), those who reported multiple racial or ethnic groups (mean = 4.66, SD = 0.67), and White adults (mean = 4.61, SD = 0.73) after Bonferroni corrections (*p*’s < 0.02, < 0.03, < 0.003; respectively).

### Rates of Reliable Improvement and Recovery

Table 3 provides estimates of reliable improvement and recovery in either anxiety or depression symptoms across race and ethnicity. Across racial and ethnic groups, differences in the prevalence of reliable improvement and recovery *χ*^2^ (5) = 16.51, *p* = 0.01, φ = 0.06, as well as the likelihood of experiencing reliable improvement or recovery *χ*^2^ (5) = 11.44, *p* = 0.04, φ = 0.05 met the threshold for statistical significance, but φ coefficients suggested that the observed cross-group differences were very small. More specifically, across racial and ethnic groups, the prevalence of reliable improvement or recovery in anxiety or depression symptoms ranged from 83.54 to 90.02%. Meanwhile, across racial and ethnic groups, the prevalence of reliable improvement and recovery in anxiety or depression ranged from 66.46 to 76.04%. In post-hoc tests, there were no statistically significant differences in reliable improvement and/or recovery outcomes between any two racial or ethnic groups after Bonferroni corrections.

**Table 3:** Rates of reliable improvement and/or recovery in symptoms of anxiety (GAD-7) or depression (PHQ-9) across race and ethnicity, *n* (%)

	Asian or Pacific Islander	Black or African American	Hispanic or Latino	Multiple	Other	White	*χ*^2^ *p-value*
	*n* = 1785	*n* = 351	*n* = 601	*n* = 532	*n* = 158	*n* = 3065	
Reliable improvement ^a^	1479 (82.86)	273 (77.78)	504 (83.86)	420 (78.95)	120 (75.95)	2,509 (81.86)	13.34 *p* = 0.02
Recovery ^b^	1496 (83.81)	278 (79.20)	506 (84.19)	427 (80.26)	119 (75.32)	2,516 (82.09)	13.47 *p* = 0.02
Reliable improvement and recovery ^c^	1352 (75.74)	246 (70.09)	457 (76.04)	371 (69.74)	105 (66.46)	2,258 (73.67)	16.51 *p* = 0.01
Reliable improvement or recovery ^d^	1599 (89.58)	299 (85.19)	541 (90.02)	467 (87.78)	132 (83.54)	2,722 (88.81)	11.44 *p* = 0.04

*PHQ-9*: 9-item Patient Health Questionnaire. *GAD-7*: 7-item Generalized Anxiety Disorder Scale

^**a**^ Reliable improvement: ≥ 4 decrease on the final GAD-7 among those with baseline GAD-7 ≥ 8 and/or ≥ 6 decrease on the final PHQ-9 among those with baseline PHQ-9 ≥ 10

^b^ Recovery: Final GAD-7 < 8 among those with baseline GAD-7 ≥ 8 and/or final PHQ-9 < 10 among those with baseline PHQ-9 ≥ 10

^c^ Reliable Improvement and Recovery: ≥ 4 decrease on the final GAD-7 and final GAD-7 < 8 among those with baseline GAD-7 ≥ 8; and/or, ≥ 6 decrease on the final PHQ-9 and final PHQ-9 < 10 among those with baseline PHQ-9 ≥ 10

^d^ Reliable Improvement or Recovery: ≥ 4 decrease on the final GAD-7 or final GAD-7 < 8 among those with baseline GAD-7 ≥ 8; and/or, ≥ 6 decrease on the final PHQ-9 or final PHQ-9 < 10 among those with baseline PHQ-9 ≥ 10

### Racial and Ethnic Differences in Trajectories of Anxiety and Depressive Symptoms

#### Anxiety symptoms

The estimate for the fixed intercept (*b* = 11.55; 95% confidence interval [CI] = 11.41, 11.69) describes the average initial GAD-7 score for White participants (Table 4). The initial trajectory slope suggests that among White participants, GAD-7 scores decline approximately 1 unit per week (*b* =  − 1.05; 95% CI =  − 1.10, − 1.01) during the initial stage of therapy, and a significant Week × Hispanic or Latino interaction (*b* =  − 0.12; 95% CI =  − 0.23, − 0.01) indicates that participants with this identity show a stronger (more negative) initial decline in symptoms. The initial trajectories for the remaining racial and ethnic groups did not significantly differ from the reference group. The rate of change in GAD-7 symptoms attenuated over the course of treatment (Week^2^ *b* = 0.05; 95% CI = 0.04, 0.05), and the degree of attenuation did not significantly differ across race and ethnicity.

**Table 4:** Growth curve modeling results of anxiety and depressive symptoms: effects of time in treatment across race and ethnicity, *b* (95% confidence interval)

	Anxiety symptoms (GAD-7)^a^	Depression symptoms (PHQ-9)^b^
Intercept	11.55 (11.41, 11.69)***	12.69 (12.50, 12.88)***
Week	− 1.05 (− 1.10, − 1.01)***	− 1.19 (− 1.25, − 1.12)***
Week^2^	0.05 (0.04, 0.05)***	0.05 (0.05, 0.06)***
Race and ethnicity		
Asian or Pacific Islander	− 0.13 (− 0.35, 0.08)	− 0.13 (− 0.42, 0.17)
Black or African American	0.30 (− 0.12, 0.71)	0.84 (0.30, 1.38)***
Hispanic or Latino	0.45 (0.13, 0.77)***	0.27 (− 0.16, 0.69)
Multiple	0.04 (− 0.30, 0.38)	0.51 (0.06, 0.97)**
Other	− 0.09 (− 0.68, 0.50)	− 0.43 (− 1.23, 0.38)
Week * race and ethnicity		
Week * Asian or Pacific Islander	− 0.05 (− 0.13, 0.02)	− 0.15 (− 0.26, − 0.05)***
Week * Black or African American	− 0.08 (− 0.23, 0.07)	− 0.26 (− 0.45, − 0.07)***
Week * Hispanic or Latino	− 0.12 (− 0.23, − 0.005)**	− 0.17 (− 0.32, − 0.02)**
Week * Multiple	0.03 (− 0.09, 0.15)	0.05 (− 0.10, 0.21)
Week * Other	0.004 (− 0.20, 0.21)	− 0.01 (− 0.30, 0.29)
Week^2^ * race and ethnicity		
Week^2^ * Asian or Pacific Islander	0.01 (− 0.001, 0.01)	0.01 (0.004, 0.02)***
Week^2^ * Black or African American	0.01 (− 0.004, 0.02)	0.02 (0.001, 0.03)**
Week^2^ * Hispanic or Latino	0.01 (− 0.0003, 0.02)	0.01 (− 0.003, 0.02)
Week^2^* Multiple	− 0.001 (− 0.01, 0.01)	− 0.002 (− 0.01, 0.01)
Week^2^ * Other	0.01 (− 0.01, 0.02)	0.01 (− 0.02, 0.03)
Sessions last 7 days	− 0.80 (− 0.89, − 0.71)***	− 0.93 (− 1.05, − 0.81)***
Sessions last 7 days * race and ethnicity		
Sessions last 7 days * Asian or Pacific Islander	− 0.07 (− 0.22, 0.08)	0.02 (− 0.17, 0.22)
Sessions last 7 days * Black or African American	− 0.47 (− 0.77, − 0.18)***	− 0.15 (− 0.53, 0.22)
Sessions last 7 days * Hispanic or Latino	− 0.24 (− 0.46, − 0.02)**	− 0.20 (− 0.48, 0.08)
Sessions last 7 days * Multiple	0.09 (− 0.15, 0.32)	− 0.06 (− 0.36, 0.24)
Sessions last 7 days * Other	− 0.41 (− 0.82, 0.002)	− 0.10 (− 0.64, 0.44)
Sessions last 8–14 days	− 0.61 (− 0.70, − 0.52)***	− 0.69 (− 0.82, -0.57)***
Sessions last 8–14 days * race and ethnicity		
Sessions last 8–14 days * Asian or Pacific Islander	− 0.08 (− 0.23, 0.08)	0.02 (− 0.19, 0.22)
Sessions last 8–14 days * Black or African American	− 0.30 (− 0.60, 0.002)	− 0.21 (− 0.59, 0.17)
Sessions last 8–14 days * Hispanic or Latino	− 0.08 (− 0.31, 0.15)	0.07 (− 0.23, 0.37)
Sessions last 8–14 days * Multiple	0.17 (− 0.07, 0.41)	0.11 (− 0.20, 0.41)
Sessions last 8–14 days * Other	0.39 (− 0.04, 0.81)	0.14 (− 0.43, 0.72)

Reference category = White. *GAD-7*: 7-item Generalized Anxiety Disorder Scale. *PHQ-9*: 9-item Patient Health Questionnaire. ^a^ Analysis only included participants with baseline GAD-7 ≥ 8. ^b^ Analysis only included participants with baseline PHQ-9 ≥ 10. **p < 0.05; ***p < 0.01

#### Depressive symptoms

The estimate for the fixed intercept (*b* = 12.69; 95% CI = 12.50, 12.88) describes the average initial PHQ-9 score for White participants. Statistically significant Week × Asian or Pacific Islander (*b* =  − 0.15; 95% CI =  − 0.26, − 0.05), Week × Black or African American (*b* =  − 0.26; 95% CI =  − 0.45, − 0.07), and Week × Hispanic or Latino (*b* =  − 0.17; 95% CI =  − 0.32, − 0.02) interactions suggest that these participants show a steeper (more negative) initial decline in depression symptoms, relative to White participants (linear *b* =  − 1.19; 95% CI =  − 1.25, − 1.12; Table 4). The decline in PHQ-9 becomes less pronounced over the course of treatment among White participants (Week^2^ *b* = 0.05; 95% CI = 0.05, 0.06). Moreover, the presence of significant Week^2^ × Asian or Pacific Islander (*b* = 0.01; 95% CI = 0.00, 0.02) and Week^2^ × Black or African American (*b* = 0.02; 95% CI = 0.00, 0.03) interactions suggest that these participants exhibited a stronger attenuation of their symptom trajectories (relative to White Participants) over the treatment course.

### Racial and Ethnic Differences in Time-varying Covariate Effects of Session Engagement

#### Anxiety symptoms

A significant coefficient for the first-order effect of past-week sessions (*b* =  − 0.80; 95% CI =  − 0.89, − 0.71) suggests that engaging with a therapy session during the previous week was associated with lower GAD-7 scores among White participants (Table 4). In addition, significant interactions emerged for Black or African American (*b* =  − 0.47; 95% CI =  − 0.77, − 0.18) and Hispanic or Latino participants (*b* =  − 0.24; 95% CI =  − 0.46, − 0.02), indicating that participants in these categories exhibited a stronger time-varying covariate effect of engaging in a session over the prior week. The first-order coefficient for sessions last 8–14 days also emerged as significant (*b* =  − 0.61; 95% CI =  − 0.70, − 0.52), indicating that engagement with a therapy session is associated with lower anxiety symptoms more than a week later. Participants did not differ significantly in the lagged effect of engaging in a therapy session across race and ethnicity.

#### Depressive symptoms

Significant first-order effects emerged for session last 7 days (*b* =  − 0.93; 95% CI =  − 1.05, − 0.81) and session last 8–14 days (*b* =  − 0.69; 95% CI =  − 0.82, − 0.57), suggesting that engaging in therapy during the past week, and the week prior were each uniquely associated with PHQ-9 scores (Table 4). All interactions of race and ethnicity with session last 7 days and session last 8–14 days failed to reach significance.

## Discussion

This retrospective cohort analysis, conducted in a large racially and ethnically diverse sample of adults enrolled in BCT, found that anxiety and depressive symptoms significantly decreased during treatment across race and ethnicity. These declines were more pronounced among some racial and ethnic groups. That is, compared to White participants, Hispanic or Latino participants had greater initial decreases in depression and anxiety symptoms, while Black or African American and Asian or Pacific Islander participants had greater initial decreases in symptoms of depression alone, across treatment. Rates of reliable improvement or recovery in symptoms of anxiety or depression were high and ranged from 83.54 to 90.02% across racial and ethnic groups but statistically significant differences were observed (yet not between any two racial and ethnic groups in post-hoc tests). In addition, although participants completed approximately 6 therapy sessions on average, differences were statistically significant across racial and ethnic groups. Furthermore, average treatment satisfaction scores were high but cross-group score differences were statistically significant. Importantly, statistically significant differences across outcomes were generally small in magnitude and not clinically significant, thus this study provides evidence of equitable effectiveness of a culturally responsive BCT program across diverse racial and ethnic groups.

The current study makes important contributions to the culturally responsive psychotherapy and BCT evidence bases. To the authors’ knowledge, this is the first large-scale outcomes evaluation of a culturally responsive video BCT program across racial and ethnic groups. Prior meta-analyses have reported greater effects for culturally adapted psychological interventions compared to unadapted or no psychological intervention among communities of color [44, 45], including culturally-adapted internet-based psychological interventions [17]. However, unlike the BCT program evaluated in the current study, most of the interventions included in those analyses were adapted for specific racial and ethnic groups. Such interventions may have stronger outcomes than those which are sensitive to numerous cultural contexts [46]; yet, psychological interventions that are culturally sensitive to broader populations may have more beneficial outcomes than those with no adaptations [45]. In fact, the findings of statistically significant differences across racial and ethnic groups for numerous study outcomes, though small in magnitude, point to the possibility BCT that is more broadly culturally responsive may be more beneficial for some cultural contexts than others. Further research is needed to determine the comparative effectiveness of culturally responsive BCT versus BCT with no, or varying levels of, cultural responsiveness across racial and ethnic groups. Such research should also include evaluation of BCT treatment fidelity across race and ethnicity. Despite the positive outcomes of this study, these results are not necessarily generalizable to all blended interventions, especially those that are not culturally responsive. BCT can vary by design (e.g., face-to-face versus internet module-focused BCT) [18], and the implementation strategy may also influence outcomes [47], highlighting the importance of performing outcomes evaluations of unique BCT programs, especially as such programs incorporate culturally responsive tools and expand their reach to more diverse populations. In addition, evaluating the independent effects of components of culturally responsive BCT on outcomes (e.g., culturally responsive digital tools, culturally responsive therapists) could inform the development of future programs and warrants further research.

Because digital mental health interventions could increase accessibility of care, researchers have highlighted their potential role in reducing racial and ethnic mental health disparities [13, 14]. The current study provides evidence that a large-scale BCT can be effective across communities of color. Yet, further research is needed to determine the extent to which employer-offered BCT can address mental health disparities, including evaluating additional metrics such as access and utilization rates across racial and ethnic groups [19]. The current study also found high levels of treatment satisfaction across race and ethnicity. These results extend those of a small study, which did not include race and ethnicity data, that reported high satisfaction of a video BCT program [48]. These findings are also aligned with a systematic review which found high satisfaction for tele-counseling interventions among communities of color [15]. However, the treatment satisfaction levels observed in the current study should be interpreted with caution in light of high non-response and the significant differences in final symptom severity levels between responders versus non responders. In addition, the current study did not evaluate, separately, satisfaction levels of digital tools, or the influence of satisfaction levels on clinical outcomes, both of which are important areas for future research across racial and ethnic groups. Another important area for future research is the evaluation of racial and ethnic differences in therapeutic alliance for culturally responsive BCT, and its association with outcomes.

The current study had numerous strengths. For one, this was a large racially and ethnically diverse sample. Additionally, this was a real-world evaluation that relied on measures of anxiety and depression that have been validated. However, there were several limitations. First, this study did not utilize a randomized controlled design to confirm causality, and cannot conclude that any treatment effects were attributable to the culturally responsive components of the program. Additionally, some racial and ethnic groups were under-represented in the sample and were conflated in the categories that included those who reported multiple and “Other” racial or ethnic groups. Meanwhile, a significant proportion of the sample was missing a response to the treatment satisfaction measure, and responders had significantly lower final PHQ-9 and GAD-7 scores than non-responders. However, there were no significant racial and ethnic differences in the response rate for this treatment satisfaction measure. Additionally, the treatment satisfaction measure was not validated, and the possibility of racial and ethnic differences in its validity cannot be ruled out. Another limitation which may have biased study findings was missing data: 11.4% of the eligible sample was excluded from the final sample, a large proportion of which was excluded due to missing follow-up outcomes. The current study cannot preclude the possibility of regression to the mean effects, which is also a notable limitation. Yet, this study reports rates of reliable improvement, which attempts to account for measurement error, and in turn, regression to the mean effects [39, 49]. Finally, because this program was an employer-provided benefit available to employees and their dependents and the sample largely reported high levels of education as well as good or fair financial status, these results may not be generalizable to other populations.

In conclusion, it is important that large-scale digital and blended mental health interventions evaluate their ability to provide effective and equitable care across diverse racial and ethnic groups. This study demonstrated that a blended care program employing culturally responsive approaches can be beneficial for anxiety and depression outcomes across diverse racial and ethnic groups. Further research is needed to evaluate longer term outcomes of BCT across race and ethnicity. Finally, because culturally responsive care extends beyond race and ethnicity, it is important to take additional cultural and contextual factors into consideration when designing and evaluating outcomes of future blended interventions for diverse populations.

## Supplementary Information

Below is the link to the electronic supplementary material.

Supplementary file1 (PDF 114 KB)

## Data Availability

Because the BCT program is offered as a mental health benefit by employers, study data cannot be shared.

## Code Availability

Because the study code was generated by a commercial organization, it is proprietary and cannot be shared.
