# Supplementary material for: Blended Care Therapy for Depression and Anxiety: Outcomes across Diverse Racial and Ethnic Groups
Source: J Racial Ethn Health Disparities. 2022 Dec 2;10(6):2731–43. doi: 10.1007/s40615-022-01450-z (PMC9717563; doi:10.1007/s40615-022-01450-z)
Supplement: Supplementary file 1 — Supplementary file1 (PDF 114 KB) [file 40615_2022_1450_MOESM1_ESM.pdf]

Supplemental Table S1. Growth Curve Modeling Results of Anxiety Symptoms (GAD-7), b (95% Confidence Interval)

	**Final Model**	**Final Model + Age & Gender**
Intercept	11.55 (11.41, 11.69)<br>t = 163.08***	11.53 (11.39, 11.67)<br>t = 160.37***
Week	-1.05 (-1.10, -1.01)<br>t = -45.04***	-1.05 (-1.10, -1.01)<br>t = -45.04***
Week^2	0.05 (0.04, 0.05)<br>t = 26.02***	0.05 (0.04, 0.05)<br>t = 26.01***
Sessions last 7 days	-0.80 (-0.89, -0.71)<br>t = -17.46***	-0.80 (-0.89, -0.71)<br>t = -17.46***
Sessions last 8-14 days	-0.61 (-0.70, -0.52)<br>t = -12.76***	-0.61 (-0.70, -0.52)<br>t = -12.76***
Gender (Female)		0.07 (-0.01, 0.15)<br>t = 1.82*
Age		-0.001 (-0.01, 0.01)<br>t = -0.20
Asian or Pacific Islander	-0.13 (-0.35, 0.08)<br>t = -1.20	-0.14 (-0.36, 0.08)<br>t = -1.24
Black or African American	0.30 (-0.12, 0.71)<br>t = 1.41	0.29 (-0.12, 0.70)<br>t = 1.37
Hispanic or Latino	0.45 (0.13, 0.77)<br>t = 2.75***	0.45 (0.13, 0.77)<br>t = 2.74***
Multiple	0.04 (-0.30, 0.38)<br>t = 0.21	0.03 (-0.32, 0.37)<br>t = 0.15
Other	-0.09 (-0.68, 0.50)<br>t = -0.29	-0.08 (-0.67, 0.51)<br>t = -0.26

Week * Asian or Pacific Islander	-0.05 (-0.13, 0.02) t = -1.39	-0.05 (-0.13, 0.02) t = -1.38
Week * Black or African American	-0.08 (-0.23, 0.07) t = -1.10	-0.08 (-0.23, 0.07) t = -1.09
Week *Hispanic or Latino	-0.12 (-0.23, -0.005) t = -2.04**	-0.12 (-0.23, -0.005) t = -2.05**
Week * Multiple	0.03 (-0.09, 0.15) t = 0.50	0.03 (-0.09, 0.15) t = 0.50
Week * Other	0.004 (-0.20, 0.21) t = 0.04	0.004 (-0.20, 0.21) t = 0.04
$Week^2$ * Asian or Pacific Islander	0.01 (-0.001, 0.01) t = 1.79*	0.01 (-0.001, 0.01) t = 1.79*
$Week^2$ * Black or African American	0.01 (-0.004, 0.02) t = 1.26	0.01 (-0.004, 0.02) t = 1.26
$Week^2$ * Hispanic or Latino	0.01 (-0.0003, 0.02) t = 1.88*	0.01 (-0.0003, 0.02) t = 1.89*
$Week^2$ * Multiple	-0.001 (-0.01, 0.01) t = -0.13	-0.001 (-0.01, 0.01) t = -0.13
$Week^2$ * Other	0.01 (-0.01, 0.02) t = 0.71	0.01 (-0.01, 0.02) t = 0.71
Sessions last 7 days * Asian or Pacific Islander	-0.07 (-0.22, 0.08) t = -0.90	-0.07 (-0.22, 0.08) t = -0.91
Sessions last 7 days * Black or African American	-0.47 (-0.77, -0.18) t = -3.15***	-0.47 (-0.77, -0.18) t = -3.14***
Sessions last 7 days*x Hispanic or Latino	-0.24 (-0.46, -0.02) t = -2.15**	-0.24 (-0.46, -0.02) t = -2.15**
Sessions last 7 days * Multiple	0.09 (-0.15, 0.32)	0.09 (-0.15, 0.32)

	t = 0.74	t = 0.74
Sessions last 7 days * Other	-0.41 (-0.82, 0.002)	-0.41 (-0.82, 0.002)
	t = -1.95*	t = -1.95*
Sessions last 8-14 days * Asian or Pacific Islander	-0.08 (-0.23, 0.08)	-0.08 (-0.23, 0.08)
	t = -0.98	t = -0.98
Sessions last 8-14 days * Black or African American	-0.30 (-0.60, 0.002)	-0.30 (-0.60, 0.001)
	t = -1.95*	t = -1.95*
Sessions last 8-14 days * Hispanic or Latino	-0.08 (-0.31, 0.15)	-0.08 (-0.31, 0.15)
	t = -0.71	t = -0.71
Sessions last 8-14 days * Multiple	0.17 (-0.07, 0.41)	0.17 (-0.07, 0.41)
	t = 1.41	t = 1.41
Sessions last 8-14 days * Other	0.39 (-0.04, 0.81)	0.39 (-0.04, 0.81)
	t = 1.78*	t = 1.78*
**Log Likelihood**	-106,305.60	-106,303.90
**Akaike Inf. Crit.**	212,687.10	212,687.80
**Bayesian Inf. Crit.**	213,013.80	213,031.70

Notes. Reference category=White. GAD-7: 7-item Generalized Anxiety Disorder Scale. Analysis only included participants with baseline GAD-7≥8. *p<0.1; **p<0.05; ***p<0.01

Supplemental Table S2. Growth Curve Modeling Results of Depression Symptoms (PHQ-9), b (95% Confidence Interval)

	**Final Model**	**Final Model + Age & Gender**
Intercept	12.69 (12.50, 12.88)	12.69 (12.49, 12.88)
	t = 128.21***	t = 126.32***
Week	-1.19 (-1.25, -1.12)	-1.19 (-1.25, -1.12)
	t = -36.41***	t = -36.40***
Week^2	0.05 (0.05, 0.06)	0.05 (0.05, 0.06)
	t = 20.87***	t = 20.86***
Sessions last 7 days	-0.93 (-1.05, -0.81)	-0.93 (-1.05, -0.81)
	t = -15.08***	t = -15.08***
Sessions last 8-14 days	-0.69 (-0.82, -0.57)	-0.69 (-0.82, -0.57)
	t = -10.88***	t = -10.88***
Gender (Female)		0.001 (-0.11, 0.11)
		t = 0.01
Age		0.002 (-0.01, 0.01)
		t = 0.29
Asian or Pacific Islander	-0.13 (-0.42, 0.17)	-0.12 (-0.42, 0.18)
	t = -0.85	t = -0.80
Black or African American	0.84 (0.30, 1.38)	0.84 (0.30, 1.38)
	t = 3.04***	t = 3.04***
Hispanic or Latino	0.27 (-0.16, 0.69)	0.27 (-0.16, 0.70)
	t = 1.22	t = 1.24
Multiple	0.51 (0.06, 0.97)	0.52 (0.06, 0.98)
	t = 2.22**	t = 2.24**
Other	-0.43 (-1.23, 0.38)	-0.42 (-1.23, 0.39)
	t = -1.03	t = -1.03
Week * Asian or Pacific Islander	-0.15 (-0.26, -0.05)	-0.15 (-0.26, -0.05)
	t = -2.81***	t = -2.81***
Week * Black or African American	-0.26 (-0.45, -0.07)	-0.26 (-0.45, -0.07)

	t = -2.63***	t = -2.62***
Week * Hispanic or Latino	-0.17 (-0.32, -0.02)	-0.17 (-0.32, -0.02)
	t = -2.21**	t = -2.21**
Week * Multiple	0.05 (-0.10, 0.21)	0.05 (-0.10, 0.21)
	t = 0.68	t = 0.68
Week * Other	-0.01 (-0.30, 0.29)	-0.01 (-0.30, 0.29)
	t = -0.05	t = -0.05
$Week^2$ * Asian or Pacific Islander	0.01 (0.004, 0.02)	0.01 (0.004, 0.02)
	t = 2.95***	t = 2.96***
$Week^2$ * Black or African American	0.02 (0.001, 0.03)<br>t = 2.15**	0.02 (0.001, 0.03)<br>t = 2.15**
$Week^2$ * Hispanic or Latino	0.01 (-0.003, 0.02)<br>t = 1.45	0.01 (-0.003, 0.02)<br>t = 1.45
$Week^2$ * Multiple	-0.002 (-0.01, 0.01)<br>t = -0.39	-0.002 (-0.01, 0.01)<br>t = -0.39
$Week^2$ * Other	0.01 (-0.02, 0.03)<br>t = 0.56	0.01 (-0.02, 0.03)<br>t = 0.56
Sessions last 7 days * Asian or Pacific Islander	0.02 (-0.17, 0.22)<br>t = 0.23	0.02 (-0.17, 0.22)<br>t = 0.23
Sessions last 7 days * Black or African American	-0.15 (-0.53, 0.22)<br>t = -0.80	-0.15 (-0.53, 0.22)<br>t = -0.80
Sessions last 7 days * Hispanic or Latino	-0.20 (-0.48, 0.08)<br>t = -1.38	-0.20 (-0.48, 0.08)<br>t = -1.38
Sessions last 7 days * Multiple	-0.06 (-0.36, 0.24)	-0.06 (-0.36, 0.24)
	t = -0.39	t = -0.39

Sessions last 7 days * Other	-0.10 (-0.64, 0.44)	-0.10 (-0.64, 0.44)
	t = -0.37	t = -0.37
Sessions last 8-14 days * Asian or Pacific Islander	0.02 (-0.19, 0.22)	0.02 (-0.19, 0.22)
	t = 0.16	t = 0.16
Sessions last 8-14 days * Black or African American	-0.21 (-0.59, 0.17)	-0.21 (-0.59, 0.17)
	t = -1.09	t = -1.09
Sessions last 8-14 days * Hispanic or Latino	0.07 (-0.23, 0.37)	0.07 (-0.23, 0.37)
	t = 0.46	t = 0.46
Sessions last 8-14 days * Multiple	0.11 (-0.20, 0.41)	0.11 (-0.20, 0.42)
	t = 0.69	t = 0.69
Sessions last 8-14 days * Other	0.14 (-0.43, 0.72)	0.14 (-0.43, 0.72)
	t = 0.49	t = 0.49
**Log Likelihood**	-72,153.14	-72,153.10
**Akaike Inf. Crit.**	144,382.30	144,386.20
**Bayesian Inf. Crit.**	144,693.20	144,713.50

Notes. Reference category=White. PHQ-9: 9-item Patient Health Questionnaire. Analysis only included participants with baseline PHQ-9≥10. *p<0.1; **p<0.05; ***p<0.01
